# Acute effects of tonic motor activation (TOMAC) on sleep in adults with medication-refractory restless legs syndrome

**DOI:** 10.1093/sleepadvances/zpag060

**Published:** 2026-06-11

**Authors:** Stephanie K Rigot, Erik K St. Louis, Fiona C Baker, Hussein Alawieh, Haramandeep Singh, Joseph Ojile, Jatin Tekchandani, Viktoriia Kolotovska, Bahman Adlou, Jonathan D Charlesworth

**Affiliations:** Noctrix Health, Inc., Pleasanton, CA, United States; Division of Pulmonary, Critical Care, Allergy, and Sleep Medicine, Departments of Neurology and Medicine, Mayo Sleep Behavior and Neurophysiology Research Laboratory, Mayo Center for Sleep Medicine, Rochester, MN, United States; Center for Health Sciences, SRI International, Menlo Park, CA, United States; Noctrix Health, Inc., Pleasanton, CA, United States; Sleep Medicine Specialists of California, San Ramon, CA, United States; Clayton Sleep Institute, St. Louis, MO, United States; Noctrix Health, Inc., Pleasanton, CA, United States; Noctrix Health, Inc., Pleasanton, CA, United States; Noctrix Health, Inc., Pleasanton, CA, United States; Noctrix Health, Inc., Pleasanton, CA, United States

**Keywords:** restless legs syndrome, peripheral nerve stimulation, sleep disorder, polysomnography, periodic limb movements, neuromodulation, electrical stimulation therapy

## Abstract

**Study Objectives:**

Tonic motor activation (TOMAC) delivers high-frequency electrical stimulation to the common peroneal nerve to reduce restless legs syndrome symptoms and improve subjective sleep quality. This cross-sectional analysis provides the first objective evaluation of the wake–sleep transition and an exploration of sleep parameter and leg movement changes following mid-sleep TOMAC sessions during polysomnography.

**Methods:**

Twenty participants with medication-refractory restless legs syndrome completed two polysomnographies and activated 30-min TOMAC sessions as needed when awakening mid-sleep with restless legs syndrome symptoms. Objective sleep parameters including sleep reinitiation latency, architecture, and hourly indices of periodic leg movements during sleep (PLMS) and PLMS associated with arousal were compared between the sleep periods before and after activating mid-sleep TOMAC sessions.

**Results:**

Sleep reinitiation occurred during the 30-min TOMAC session for 24 of 29 (82.8%) mid-sleep sessions, with a median latency of 6.7 (range = 0.9–45.9) min following TOMAC activation. The latency from TOMAC activation to sleep reinitiation was shorter in participants who activated TOMAC within 10 min of awakening compared to those who waited longer (*p* = 0.016). There was a linear increase in the proportion of delta and a decrease in alpha electroencephalogram (EEG) frequency-band power after activating mid-sleep TOMAC (both *p* < 0.001), indicating the transition towards deeper sleep. Comparing sleep intervals before and after activating mid-sleep TOMAC, PLMS/hour decreased by 47.9% (*p* = 0.042) and PLMS associated with arousal/hour decreased by 70.8% (*p* = 0.004).

**Conclusions:**

TOMAC was compatible with sleep; rapid sleep reinitiation and a transition to deeper sleep were observed during TOMAC stimulation. These data also provide proof-of-concept evidence that PLMS may decrease following activation of mid-sleep TOMAC.

**Clinical Trial Details:**

This study was preregistered on January 31, 2022 ClinicalTrials.gov with name “Noninvasive Peripheral Nerve Stimulation for Medication-Naive and Medication-Refractory RLS” and number NCT05214963 at https://clinicaltrials.gov/study/NCT05214963.

**Statement of Significance**

Tonic motor activation (TOMAC) has demonstrated efficacy in treating restless legs syndrome (RLS) symptoms and improving subjective sleep quality in adults with moderate-to-severe medication-refractory RLS. However, to our knowledge, there has been no previous evidence for TOMAC effects on objective sleep parameters. Participants were able to rapidly reinitiate sleep during mid-sleep TOMAC stimulation and often exhibited transitions to more restorative sleep stages. Periodic leg movements during sleep (PLMS) and associated arousals were also reduced following TOMAC activation. This study demonstrates that TOMAC stimulation is compatible with sleep. Furthermore, our data provide proof-of-concept evidence motivating future research to determine if TOMAC causes a reduction in PLMS.

## Introduction

Restless legs syndrome (RLS) is a chronic sleep-related movement disorder characterized by an uncomfortable urge to move the legs that is most prominent and severe in the evening and periods of rest or inactivity [1]. RLS is prevalent in 5–10% of the global population and 2–3% of Western populations have clinically significant RLS symptoms causing substantial distress or impairment to social, occupational, educational, or other important activities of daily living, as well as deleterious impact on sleep quality and quality of life [1–3]. Over 75% of patients with RLS report having sleep-related symptoms including interrupted sleep, an inability to fall or stay asleep, and insufficient sleep duration [2]. In the short term, sleep disruption can result in an increased stress response, mood disorders, and cognitive, memory, and performance deficits. Long-term risks associated with sleep disruption include hypertension, dyslipidemia, cardiovascular disease, metabolic syndrome, and colorectal cancer [4–6]. Consequently, effective management of RLS-related sleep disruption is a medical necessity to mitigate these substantial health implications.

The tonic motor activation (TOMAC; Noctrix Health, Inc., Pleasanton, CA, USA) system is a wearable, nonpharmacological neuromodulation device that is FDA De Novo granted in the United States to reduce symptoms and improve sleep for adults with primary moderate-to-severe, medication-refractory RLS. The TOMAC device is worn bilaterally below each knee over the common peroneal nerve and delivers high-frequency electrical stimulation for 30-min sessions. Evidence supports that the mechanism of action of TOMAC is the selective activation of afferent fibers in the peroneal nerve that engage neural pathways similar to those activated by voluntary movements such as foot flexion or walking [7]. TOMAC is associated with noticeable sensations related to the activation of these proprioceptive fibers. Patients who awaken with RLS symptoms during the sleep period (“mid-sleep”) are instructed to activate a 30-min session of TOMAC at that time [8]. Although TOMAC is associated with subjective improvements in sleep across weeks of use [9], to our knowledge, there has been no previous demonstration using polysomnography (PSG) to objectively confirm that patients can fall asleep during TOMAC stimulation. Therefore, the aim of this study was to objectively evaluate the wake–sleep transition and sleep reinitiation latency following mid-sleep TOMAC activation. A secondary, exploratory goal was to investigate objective sleep parameters (sleep depth, movements, and arousals) in the subsequent period of sleep following TOMAC in comparison to sleep prior to activation.

## Materials and methods

The present work is a post hoc analysis of previously unpublished data from a secondary study primarily following a three-site randomized controlled trial (ClinicalTrials.gov number NCT05214963) where the principal study outcomes have been previously published [10]. The study protocol and informed consent were approved by a central Institutional Review Board (WCG Institutional Review Board), and all procedures were conducted in accordance with the International Conference on Harmonization guidelines on good clinical practice and with the 1964 Helsinki Declaration and its later amendments or comparable ethical standards. Informed consent was obtained from all individual participants included in the study.

### Participants

Eligible participants were between 22 and 89 years of age and had a diagnosis of primary, moderate-to-severe, medication-refractory RLS. Moderate-to-severe RLS was defined by the International RLS Study Group diagnostic criteria [1], International RLS Study Group Rating Scale (IRLS) total score ≥ 15 [11], and RLS symptoms interfering with sleep ≥4 nights per week. Medication-refractory RLS was defined as current or past failure of efficacy and/or tolerability of one or more dopamine agonist or alpha-2-delta ligand (i.e. gabapentin, gabapentin enacarbil, pregabalin) medications [9, 10, 12]. Participants were required to experience their most significant RLS symptoms in their lower legs and/or feet that occurred at or following bedtime. Participants also had to possess necessary technology to complete electronic questionnaires and communications and agree to not make major lifestyle changes in their bedtime, diet, exercise, or career during the study. All participants had previous experience using TOMAC through their participation in previously published trials [9, 10, 12].

Key exclusion criteria included receiving an inconsistent dose or schedule of medications likely to affect RLS symptoms (e.g. RLS medications, antidepressants, sleep medications, sedative antihistamines), a change in medications likely to affect RLS symptoms in the past month, a primary sleep disorder other than RLS that significantly interfered with sleep (as confirmed by the first PSG; see details in Study Procedures and Treatment section), RLS known to be caused by another condition, severe peripheral neuropathy, skin conditions affecting the application site, known allergy to device materials, active medical implant, epilepsy, dialysis, iron-deficient anemia, pregnancy, recent major surgery, or a typical bedtime outside the range of technician availability. An independent medical monitor was responsible for adjudicating adverse events (AEs).

### Study procedures and treatment

**Figure 1:**
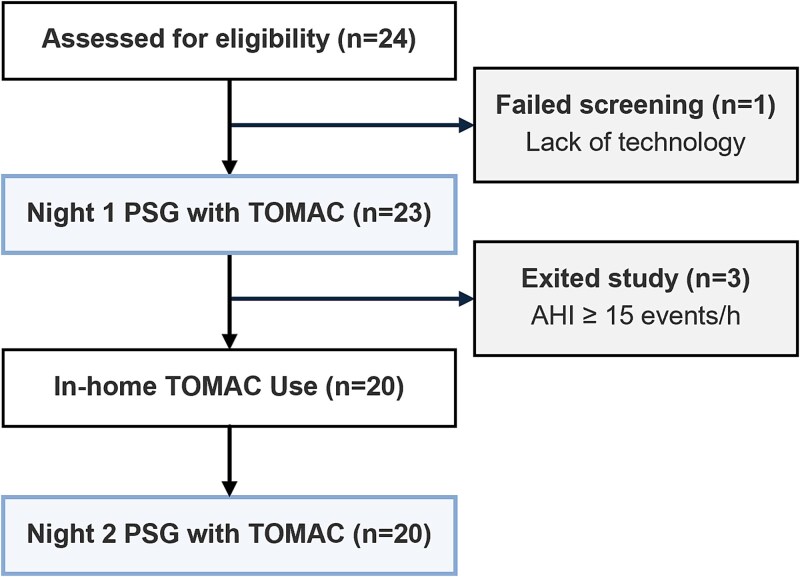
Participant disposition per PSG. Flowchart with gray boxes describing where participants exited the study and blue boxes describing PSG trials recorded by each participant.

All participants provided informed consent, were screened for eligibility, completed baseline procedures (demographic information, medical and medication history, confirmation of titrated TOMAC settings), and then underwent recording of a first in-laboratory PSG (Figure 1). Participants had the stimulation intensity for TOMAC titrated using a previously described procedure to determine the maximum stimulation intensity that was subjectively compatible with sleep within a range of 15-40 mA [7, 10, 13]. After completing the first PSG, participants with evidence of another primary sleep disorder (e.g. moderate or severe obstructive sleep apnea [OSA] having apnea-hypopnea index [AHI] ≥15/h with use of continuous positive airway pressure [CPAP], if applicable) were excluded from further participation in the study. The same participants used TOMAC at home as needed for 7–14 days and then completed a second PSG.

For all TOMAC PSG recordings, participants wore the TOMAC system on each leg throughout the sleep period. Participants were instructed to initiate TOMAC stimulation sessions whenever RLS symptoms were present at bedtime (designated hereafter as bedtime sessions) and/or during awakenings after initial sleep onset (designated mid-sleep sessions). TOMAC stimulation terminated automatically after 30 min. Depending on stimulation intensity and bioimpedance, the rechargeable battery would allow patients to complete between 1 and 4 TOMAC sessions during one evening.

### Polysomnographic sleep recording

Each PSG was recorded overnight in a sleep laboratory. Efforts were made to accommodate participants’ self-selected bedtimes and wake-up times based upon their usual routine. PSG recording was performed according to the American Academy of Sleep Medicine (AASM) guidelines [14] using Philips Alice 6 equipment at two sites and Compumedics Grael 4K at one site. The following parameters were included in the PSG study (all ≥200 Hz sampling rate): electroencephalogram (EEG, 6 channels [frontal, central, occipital] each referenced to contralateral mastoids), electrooculogram, submental electromyogram (EMG), electrocardiography, pulse oximetry, and bilateral tibialis anterior EMG, as well as thoracic and abdominal piezoelectric bands, nasal cannula and thermistor, snoring sensor, and position sensor. EMG signals were bandpass filtered at 10–100 Hz with a notch filter at 60 Hz. Signal quality and impedances were monitored throughout the recording. Participants who typically used CPAP for OSA utilized CPAP during all PSG recordings.

### PSG scoring and analysis

#### Sleep events and periodic leg movements during sleep scoring

A single registered polysomnographic technologist (RPGST) scored all PSG recordings in Philips Sleepware G3 software for all 30-s epochs according to AASM guidelines [14]. The RPSGT was blinded to TOMAC use during the PSG but was aware that TOMAC could be utilized during the night. A desaturation of 4% was used in the scoring of respiratory events. All leg movements during sleep (LMS) with durations of 0.5–10 s were manually identified for both legs by the RPGST and a custom algorithm was used to classify each candidate LMS as a periodic leg movement during sleep (PLMS) following the AASM criteria, including: a minimum of four consecutive LMS with movement onset intervals (intermovement intervals) between 5 and 90 s, LMS on different legs separated by <5 s between movement onsets counted as a single LMS, and LMS within 0.5 s of a respiratory event excluded. PLMS associated with arousal (PLMA) were identified when there was <0.5 s between the end of one PLMS or arousal event and the onset of the other [14]. Only movements during sleep are presented. Full-night PSG parameters were calculated following the AASM guidelines [14].

#### TOMAC session analysis

TOMAC sessions with durations of <20 min were excluded from the analysis as these may not be representative of the full effects of the therapy (*n* = 3). Mid-sleep TOMAC sessions (*n* = 29) were the primary focus of this analysis, however, bedtime TOMAC sessions (*n* = 7) were also evaluated as a supplementary analysis to assure similar features for sleep compatibility as for mid-sleep TOMAC sessions. Only mid-sleep TOMAC sessions were used in the exploration of changes in sleep dynamics before and following TOMAC activation, given the absence of a comparative sleep period recorded prior to bedtime TOMAC sessions.

TOMAC and PSG times were aligned based on technician documentation, PSG and TOMAC system timestamps, and visual comparison of limb movement signals recorded by the TOMAC system (not further analyzed or discussed herein) and the PSG tibialis anterior EMG. For sleep compatibility analyses, time was normalized to TOMAC activation. When comparing the period of sleep before and after TOMAC activation, sleep periods had to have a minimum duration of 5 min immediately preceding or following the awakening when TOMAC was activated, respectively. All group-level time-series analyses were averaged across all TOMAC sessions for each 30-s epoch (e.g. mean of the number of PLMS across all applicable TOMAC sessions for each normalized 30-s epoch). Sleep efficiency was defined as the proportion of 30-s epochs across all TOMAC sessions that were scored as sleep as opposed to wake. To provide a comparable reference to the TOMAC session analyses, full-night PSG parameters were weighted by the number of TOMAC sessions per night.

Time series plots were smoothed using a moving average filter over the previous 3 min. TOMAC sessions were only included in the time series plots and comparisons if they had unique data from the entire necessary duration (e.g. had ≥15 min of sleep after the completion of a previous TOMAC session). Comparisons of rates of movement, arousal, and respiratory measures were made between the 15 min of sleep prior to activating TOMAC (“before”) and 30 min of sleep after starting TOMAC (“after,” *n* = 24 sessions had sufficient duration before TOMAC) to align with the 30-min duration of TOMAC sessions and to maximize the sample size. Paired samples *t*-tests and Mann–Whitney U tests were used as appropriate with the level of significance set at *p* < 0.05. Linear regression was used to evaluate for predictors of latency from mid-sleep TOMAC activation to sleep reinitiation.

Three sub-group analyses were performed to evaluate differences between participant attributes, TOMAC session characteristics, and sleep architecture for various conditions. To evaluate for factors that may have affected sleep reinitiation during mid-sleep TOMAC sessions, sub-group analyses were performed to compare (1) sessions involving successful or unsuccessful sleep reinitiation during TOMAC, and (2) sessions involving TOMAC activation within 10 min of awakening compared to sessions in which participants waited >10 min to activate the TOMAC session after awakening. A 10-min threshold was determined by applying 95% confidence intervals for durations between awakening and TOMAC activation during all mid-sleep TOMAC sessions (9.8 min). In Appendix S3, a third sensitivity analysis was conducted to compare the first and second PSG nights, since the first PSG night was included in the analysis and first night effects could mediate differences in sleep architecture compared to subsequent PSG recording nights [15].

#### E‌EG spectral analysis

Periods of continuous EEG signals with technically inadequate data were automatically detected and excluded. Technically valid EEG signals were then filtered between 0.3 and 30 Hz using a zero-phase FIR filter to remove slow drifts and high-frequency noise. The filtered EEG was segmented into overlapping 30-s epochs with a 15-s step, providing high temporal resolution for time-frequency analysis. All presented analyses were performed on the occipital channel (O2-M1), selected for its stability across participants.

Spectral decomposition was performed using a multi-taper approach (Discrete Prolate Spheroidal Sequences) to reduce variance and improve frequency resolution [16]. For each 30-s epoch, seven tapers (time-bandwidth product NW = 4) were applied, and the resulting spectra were averaged. Power in EEG frequency bands—delta (1–4 Hz), theta (4–8 Hz), alpha (8–12 Hz), and sigma (12–15 Hz)—was calculated by averaging the multi-taper power spectral density within each band. Epoch-wise band-power time series were smoothed using a 3-min moving average. To account for individual differences, relative powers were computed by normalizing band powers to the total power per epoch for group comparisons. Pearson correlations were used to evaluate trends over time.

## Results

### Participants

Twenty-three participants were enrolled between March 16, 2022 and August 10, 2023, of which 16 participants were included in the analyses (Figure 1). The remaining seven participants were excluded for the following reasons: moderate–severe OSA detected during PSG night 1 (*n* = 3) and failure to activate any TOMAC sessions during either PSG night (*n* = 4). Fourteen participants activated a total of 31 mid-sleep TOMAC sessions, of which, 29 sessions from 20 unique PSG nights were included in the primary mid-sleep TOMAC session analysis and the remaining two mid-sleep TOMAC sessions were excluded due to durations <20 min. Further details are described in Appendix S1 and Tables S1–S3**.** Participant characteristics are shown in Table 1**.**

**Table 1:** Participant demographics at enrollment

	**Participants with mid-sleep TOMAC**
Number of participants	14
Age (y), mean (SD)	55.6 (10.2)
% Female (*n*)	64.3% (9)
Years since RLS symptom onset, mean (SD)	23.7 (14.3)
Years since RLS diagnosis, mean (SD)	11.5 (7.7)
Years since RLS starting prescription RLS medication, mean (SD)	11.5 (8.4)
OSA diagnosis, % (*n*)	29% (4)
Refractory RLS medications, % (*n*)	
Dopamine agonist	78.6% (11)
Alpha-2-delta ligand	50.0% (7)
Both	28.6% (4)
Current RLS medications, % (*n*)	
None	28.6% (4)
Dopamine agonist	57.1% (8)
Alpha-2-delta ligand	21.4% (3)
Benzodiazepines	0% (0)
Opioids	0% (0)
Total IRLS score, mean (SD)	25.4 (6.2)

Demographics at enrollment for participants included in the primary analysis of mid-sleep TOMAC sessions activated after initial sleep onset (*n* = 5 participants also ran a bedtime TOMAC session).

### Sleep initiation during TOMAC

**Figure 2:**
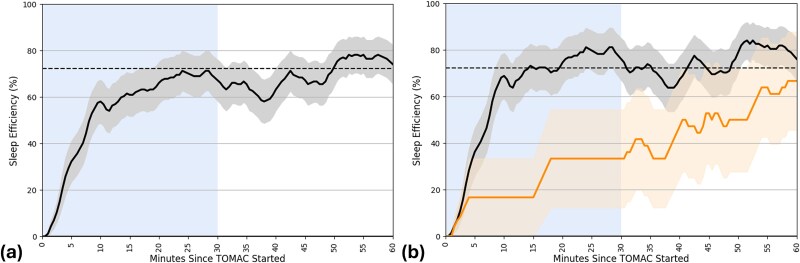
Reinitiation of sleep following mid-sleep TOMAC activation. Sleep efficiency for (a) all mid-sleep TOMAC sessions (*n* = 29 sessions) and (b) comparison between mid-sleep TOMAC sessions started ≤10 min after awakening (black, *n* = 23 sessions) and sessions started >10 min after awakening (orange, *n* = 6 sessions). The programmed 30-min TOMAC session duration is shaded blue. Sleep efficiency weighted average for the full night (72.3%) is shown by the dashed line for reference. Shaded error bands correspond to ± SEM.

Sleep reinitiation was observed during 82.8% of mid-sleep TOMAC sessions (24 of 29) and among 92.9% of participants (13 of 14). Median latency from TOMAC activation to sleep reinitiation was 6.7 min (Table 2). Average TOMAC stimulation intensity was 25.5 ± 5.3 mA, within the range of 15–40 mA tested in prior randomized clinical trials [9, 10]. Within 23 min after activating TOMAC, mean sleep efficiency exceeded the average sleep efficiency for the night (Figure 2a) and exceeded the sleep efficiency prior to activating TOMAC (Figure S1). We also observed that sleep initiation occurred during TOMAC for 71.4% of the bedtime TOMAC sessions (5 of 7, Appendix S2, these bedtime sessions were not included in any of the subsequent analyses).

**Table 2:** Compatibility of mid-sleep TOMAC sessions with sleep reinitiation

	**All mid-sleep TOMAC sessions**	**Sleep reinitiation during the TOMAC session**		**TOMAC session activation latency after awakening**	
		**Sleep reinitiation**	**Remained awake**	**≤10 min**	**>10 min**
Latency from TOMAC activation to sleep onset (minutes)		***p* < 0.001**		***p* = 0.016**	
Median (Range)	6.7 (0.9−45.9)	5.4 (0.9−22.1)	41.3 (30.5−45.9)	5.7 (0.9−41.1)	34.2 (1.4−45.9)
Mean (SD)	12.1 (14.0)	6.4 (5.7)	39.7 (5.9)	7.7 (9.0)	29.0 (17.4)
Latency from awakening to TOMAC activation (minutes)		***p* = 0.001**		***p* < 0.001**	
Median (Range)	3.0 (0.1−90.9)	2.5 (0.1−15.6)	16.2 (5.4−90.9)	2.5 (0.1−9.5)	15.9 (14.0−90.9)
Mean (SD)	8.6 (17.2)	3.9 (4.3)	31.4 (34.5)	2.9 (2.5)	30.4 (30.3)
Stimulation intensity (mA)		*p = 0.817*		*p *= 0.646	
Mean (SD)	25.5 (5.3)	25.5 (5.6)	25.6 (3.9)	25.3 (5.2)	26.5 (5.9)
TOMAC activation time (minutes after lights out)		*p = 0.978*		*p* = 0.581	
Mean (SD)	158.3 (104.3)	159.8 (108.3)	151.2 (93.4)	153.2 (107.6)	178.0 (97.1)

Left column: Characteristics of all mid-sleep (after sleep onset) TOMAC sessions run by all participants (*n* = 29 sessions). Middle columns: Comparison between subgroups of mid-sleep TOMAC sessions where the participants fell asleep during the 30-min TOMAC session (successful sleep reinitiation, *n* = 24 sessions) or remained awake throughout the session (*n* = 5 sessions). Right columns: Comparison between subgroups of mid-sleep TOMAC sessions where the participants activated the session within 10 min of awakening (*n* = 23 sessions) or waited longer than 10 min after awakening before activating TOMAC (*n* = 6 sessions). *p*-values < 0.05 between sub-groups are denoted in bold text.

Sleep reinitiation was more rapid for participants who activated TOMAC shortly after waking with RLS symptoms (*p* < 0.001, linear regression slope = 0.52, intercept = 7.7 min). The median latency from TOMAC activation to sleep onset was 5.7 min for sessions where TOMAC was activated within 10 min after waking versus 34.2 min for sessions where the participant waited longer than 10 min after waking to activate TOMAC (*p* = 0.016, Table 2). Furthermore, sleep efficiency throughout the 30-min TOMAC session was higher for the sessions activated within 10 min after waking (mean ± SEM = 24.0 ± 17.5% vs. 63.3 ± 10.1%, *p* = 0.046, Figure 2b). None of the following factors were predictive of latency from TOMAC activation to sleep onset (all *p* > 0.05): stimulation intensity, time of night, IRLS score, age, weight, leg circumference, years since RLS symptom onset, years since RLS diagnosis, or years since start of prescription medication for RLS.

**Figure 3:**
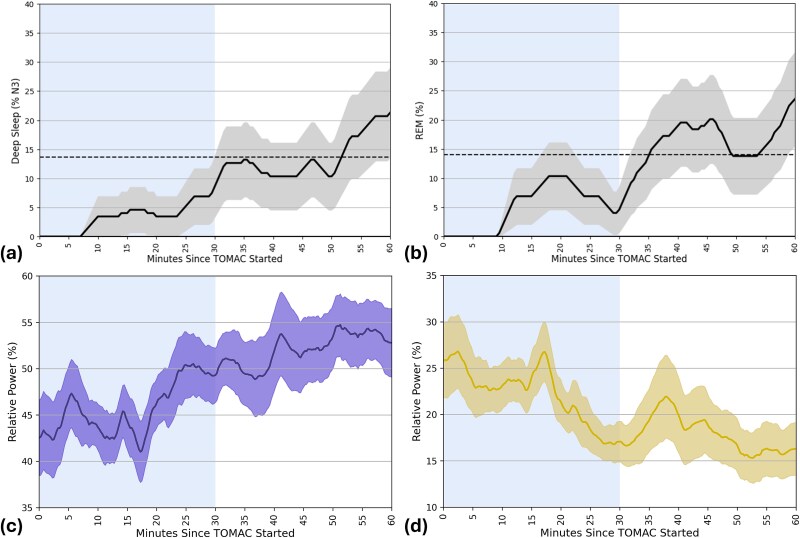
Transitions in sleep architecture following mid-sleep TOMAC activation. Top row: The proportion of mid-sleep TOMAC sessions (*n* = 29 sessions) where the 30-s epoch was scored as (a) N3 (full night average: 13.7%) and (b) REM (full night average: 14.0%) based on the scored sleep stages with the weighted average for the full night shown by the dashed line for reference. Bottom row: The averaged relative power per epoch in the (c) delta (1–4 Hz) and (d) alpha (8–12 Hz) EEG frequency bands over the 60 min since the TOMAC session was activated. For all subplots, the programmed 30-min TOMAC duration is shaded blue and shaded error bands correspond to ± SEM.

Next, we evaluated the quality of sleep following mid-sleep TOMAC activation. Participants entered N3 and rapid eye movement (REM) sleep during 13.8% (*n* = 4) and 10.3% (*n* = 3) of TOMAC sessions, respectively (Figure 3a and b). Moreover, N3 and REM percentages were similar during the 30 min of sleep after TOMAC activation and the 15- and 30-min periods of sleep preceding TOMAC activation (*p* > 0.05 for each comparison, Figure S2). Across the 60 min following TOMAC activation, EEG demonstrated a linear increase in delta (*r* = 0.92, *p* < 0.001), decrease in alpha (*r* = −0.85, *p* < 0.001, Figure 3c and d) and sigma (*r* = −0.77, *p* < 0.001), and no change in theta (*r* = 0.02, *p* = 0.805) frequency relative power.

### Leg movements, arousals, and respiratory events before and after TOMAC activation

**Figure 4:**
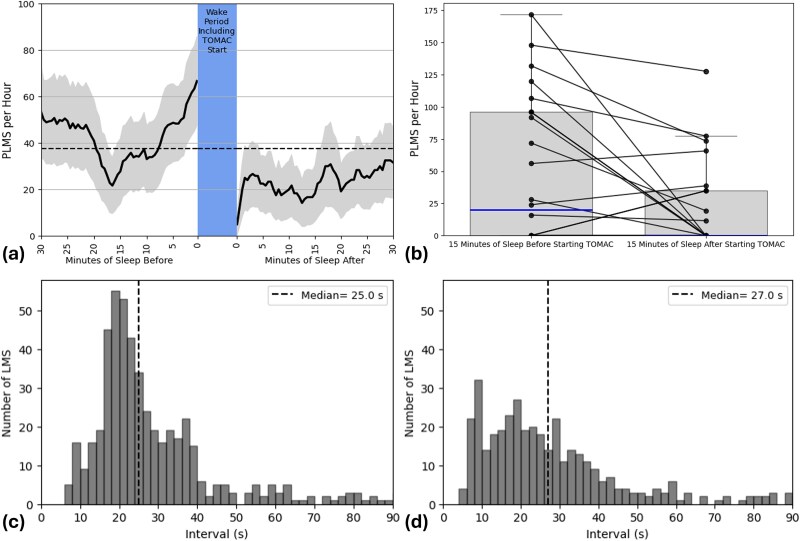
Frequency of leg movements during sleep preceding and following mid-sleep TOMAC activation. (a) Mean frequency of PLMS/h per 30-s epoch in the 30 min of sleep before and after the mid-sleep awakening when TOMAC was activated (shaded blue) with the full night average (37.6 PLMS/h) shown by a dashed line for reference and shaded error bands correspond to ± SEM. (b) Boxplot of the distribution of PLMS/h in the 15 min of sleep before and after the mid-sleep awakening, with black lines representing data for each individual mid-sleep TOMAC session. Histograms of the intermovement interval of LMS during the 30 min of sleep (c) preceding and (d) following the awakening to initiate the mid-sleep TOMAC session. *n* = 24 mid-sleep TOMAC sessions for each subplot.

Next, we evaluated leg movements before and after mid-night TOMAC session activation. Before TOMAC, PLMS frequency was elevated relative to the nightly average (Figure 4a). In the 30 min of sleep after a TOMAC activation, PLMS frequency decreased by 47.9% (*p* = 0.042). PLMS frequency exceeded the clinical threshold of 15 per hour [17, 18] before TOMAC for 54.2% of sessions (*n* = 13) compared to 33.3% (*n* = 8 sessions) after activating TOMAC (Figure 4b). PLMS remained reduced for at least 1 h after activating TOMAC (Figure S3). Other leg movement metrics showed similar trends. LMS decreased by 43.9% (*p* = 0.030) after TOMAC activation and the subset of LMS with intermovement intervals between 15 and 30 s commonly associated with RLS [19,20]- reduced by 73.8% (*p* = 0.027, Figure 4c and d).

We also evaluated events related to arousals and respirations before and after TOMAC activation. Hourly indices of total arousals (65.3% decrease, *p* = 0.001), PLMA (70.8% decrease, *p* = 0.004), and respiratory effort-related arousals (RERAs, 69.8% decrease, *p* = 0.047) all decreased following TOMAC activation (Figure S4). Arousals unassociated with PLMS were similar (*p* = 0.232) before and after TOMAC activation. Respiratory measures including apneas, hypopneas, and AHI were also similar before and after TOMAC activation (all *p* > 0.05), whereas respiratory disturbance index decreased by 69.2% (*p* = 0.019) following TOMAC.

### Safety

There were no AEs graded as 3 (severe) or higher and no serious AEs among all participants enrolled. Three device-related AEs from unique participants were reported during active TOMAC and all were graded as 1 (mild) severity: 2 of these participants had mild skin irritation (1 of which resolved with no action and 1 resolved with medication) and 1 participant noted uncomfortable or irritating sensations (which resolved with reduced treatment intensity). No device-related AEs occurred during a PSG recording. Non-device related AEs were reported by 3 participants during the in-home TOMAC period (all grade 1). No participants discontinued participation due to an AE.

## Discussion

We found that when participants with medication-refractory RLS awakened and activated TOMAC to treat their RLS symptoms, they were able to fall back asleep quickly, typically during the 30-min session of TOMAC stimulation. Furthermore, participants were often able to return to restorative sleep during TOMAC stimulation, with gradual increases in N3, REM, and delta frequency power observed. These results demonstrate that TOMAC is compatible with sleep and are consistent with previous subjective reports that TOMAC stimulation improves sleep quality [9, 10, 12]. Consistent with the TOMAC device instructions to activate a session shortly after RLS onset [8], activating TOMAC within 10 min after awakening yielded faster sleep reinitiation than waiting longer. TOMAC stimulation intensity was titrated in the same manner previously shown to evoke EMG activity [7] and reduce RLS symptoms [9, 10, 12], and the titrated intensities herein were comparable to prior clinical trials [9, 10, 12], suggesting that “full intensity” TOMAC stimulation is compatible with sleep.

Reductions in PLMS and associated arousals were observed following TOMAC activation. PLMS generally decrease throughout the time course of the night [18, 19], but a minimal decrease should have been anticipated during our short analysis window before and after TOMAC activation. PLMS are clinically significant symptoms that have been associated with increased heart rate, blood pressure, and cerebrovascular disease [18, 20–22], whereas arousals can lead to sleep fragmentation, increased sleepiness, decreased sleep efficiency, diminished psychomotor performance, cognitive decline, and cardiovascular risks [23–28]. Current standard of care to treat RLS includes alpha-2-delta ligand medications, dopamine agonists, and opioids although these pharmaceuticals may have limited efficacy in reducing PLMS or improving sleep and may have adverse effects including augmentation, impulse control disorder symptoms, somnolence, dizziness, and chemical dependence [29–33]. Most other non-pharmacological treatments for RLS have lacked consistent clinical efficacy and/or sleep compatibility [34–39]; however, TOMAC has demonstrated effectiveness to reduce RLS symptoms, improve subjective sleep quality [9, 10, 12], and is objectively compatible with sleep.

There were a number of limitations to this post hoc analysis. Most importantly, there was no control data to use for comparison, and thus we were limited to a pre/post TOMAC session analysis. Therefore, the results here demonstrate that TOMAC is compatible with sleep onset and transitions to deeper sleep stages but cannot demonstrate that TOMAC objectively improves sleep compared to no or other treatment(s). Ideally, future research should include a comparator night wherein blinded participants are instructed to activate a sham TOMAC device when they awaken with RLS symptoms (i.e. same instructions as for active TOMAC therapy). Future work should also evaluate changes to sleep after at least 4–8 weeks of TOMAC use, since the subjective effects of TOMAC on RLS improve over time [9, 12]. There were also limitations related to including two nights of PSG recording per participant; participants may have been getting accustomed to the PSG environment and equipment during the first night [15]. Whereas there were no differences in sleep architecture between the first and second PSG nights, movements and arousals were lower after TOMAC in the second night, so it is possible that the inclusion of the first PSG night resulted in more conservative findings. Our use of AASM scoring criteria may have resulted in overestimating the periodicity of leg movements and underestimating the frequency of respiratory-related leg movements relative to World Association of Sleep Medicine scoring criteria [40–42]. One participant was included who reported taking an antidepressant medication that may have affected that participant’s response to TOMAC or their PSG results; however, this effect is not likely to have substantially impacted the overall PSG findings. Other limitations of this study included the short treatment duration and small sample size.

In conclusion, TOMAC stimulation does not interfere with sleep and is compatible with rapid sleep reinitiation and transitions into restorative sleep for individuals with refractory RLS. There were no abnormalities in movements, arousals, or respiratory events in the period of sleep after the TOMAC sessions. Moreover, the activation of TOMAC sessions following awakenings during the sleep period was associated with reductions in PLMS and arousals. Future sham-controlled studies utilizing PSG will be necessary to confirm whether TOMAC therapy can reduce PLMS and arousals, and to explore the potential for TOMAC to improve objective sleep parameters.

## Supplementary Material

TOMAC_PSG_Sleep_Compatibility_Supplementary_Materials_zpag060

## Data Availability

The data underlying this article will be shared on reasonable request to the corresponding author.
