# Supplementary material for: Acute effects of tonic motor activation (TOMAC) on sleep in adults with medication-refractory restless legs syndrome
Source: Sleep Adv. 2026 Jun 11;7(3):zpag060. doi: 10.1093/sleepadvances/zpag060 (PMC13390924; doi:10.1093/sleepadvances/zpag060)
Supplement: TOMAC_PSG_Sleep_Compatibility_Supplementary_Materials_zpag060 [file tomac_psg_sleep_compatibility_supplementary_materials_zpag060.docx]

**Supplementary Material:**

**Acute Effects of Tonic Motor Activation (TOMAC) on Sleep in Adults with Medication-Refractory Restless Legs Syndrome**

Stephanie K. Rigot ^a^, Erik K. St. Louis ^b^, Fiona C. Baker ^c^,

Hussein Alawieh ^a^, Haramandeep Singh ^d^, Joseph Ojile ^e^,

Jatin Tekchandani ^a^, Viktoriia Kolotovska ^a^, Bahman Adlou ^a^, Jonathan D. Charlesworth ^a *^

^a^ Noctrix Health, Inc., 6700 Koll Center Pkwy, Suite 310, Pleasanton, CA 94566, USA

^b^ Mayo Sleep Behavior and Neurophysiology Research Laboratory, Mayo Center for Sleep Medicine, Division of Pulmonary, Critical Care, Allergy, and Sleep Medicine, Departments of Neurology and Medicine, Mayo Clinic, 200 1^st^ St SW, Rochester, MN, 55905, USA

^c^ Center for Health Sciences, SRI International, 333 Ravenswood Ave, Menlo Park, CA 94025, USA

^d^ Sleep Medicine Specialists of California, 5201 Norris Canyon Rd, Suite 120, San Ramon, CA 94583, USA

^e^ Clayton Sleep Institute, LLC, 11188 Tesson Ferry Road, Suite 100, St. Louis, MO 63123, USA

* Corresponding author, email: jcharlesworth@noctrixhealth.com

## Appendix S1: Detailed participant and TOMAC sessions information.

Twenty-four participants were screened for the study, with 15 participants (62.5%) having participated in the related randomized controlled trial [10] and 9 participants being recruited from prior trials [9,12]. One participant failed screening given lack of necessary technology and/or internet access to complete study procedures. After the 23 enrolled participants completed the first PSG, 3 were excluded from further participation due to evidence of moderate or severe OSA with AHI ≥ 15 events/h (**Table S1**). For the 20 participants who completed the remainder of the study, the duration of in-home TOMAC use prior to their second PSG with TOMAC was 7-22 days with a mean±SD of 13.4±4.2 days.

Four participants were excluded from all analyses due to not completing a TOMAC session during either of the PSG recordings (n=3 participants did not attempt a session, n=1 participant’s only TOMAC session was short duration during the PSG recording due to majority of session running prior to starting the recording). Two additional participants only ran TOMAC sessions at bedtime prior to initial sleep onset and were only included in the supplementary analysis for determination of sleep onset latency following TOMAC application (**Table S2, Appendix S2**). Fourteen participants ran at least one mid-sleep TOMAC session after initial sleep onset and were included in the primary analyses. Additionally, three individual TOMAC sessions were excluded from the analysis due to short session duration (1-18 minutes) resulting in a final sample of 29 mid-sleep TOMAC sessions during 20 PSG nights from 14 participants.

In addition to the participant characteristics in **Table 1**, no participants reported a diagnosis of insomnia and all participants with OSA that were included in the analysis used CPAP and had an AHI < 15 events/h during all PSG recordings. Among participants taking the medication, daily doses of dopamine agonists varied from 0.375 - 4 mg (n= 8 participants) and alpha-2-delta ligand medications varied from 300 - 900 mg (n=3 participants). Additionally, one participant reported taking a 15 mg daily dose of Mirtazapine (noradrenergic and specific serotonergic antidepressant) which may cause drowsiness.

Table S1: Individual participants, PSG nights with TOMAC, and TOMAC sessions included in each analysis with summary totals.

| **Partic-ipant #** | **Site #** | **Participant Included in Analyses** | | | **PSG Night with TOMAC Included in Analyses** | | | | **TOMAC Sessions Activated** | | | | | | **‡ Exclude Reason** |
| --- | --- | --- | --- | --- | --- | --- | --- | --- | --- | --- | --- | --- | --- | --- | --- |
|  |  | **Any** | **MS** | **B** | **Night #** | **Any** | **MS** | **B** | **Session Timing** | **Sess. #** | **In Any Analyses** | **Sleep During TOMAC** | **< 10-min Awake to TOMAC Start** | ***In Sleep Before/ After Analyses** |  |
| 1 | 1 | Y | Y | N | 1 | Y | Y | N | MS | 1 | Y | Y | Y | N | - |
|  |  |  |  |  |  |  |  |  | MS | 2 | Y | Y | Y | N † | - |
|  |  |  |  |  |  |  |  |  | MS | 3 | Y | Y | Y | Y | - |
|  |  |  |  |  |  |  |  |  | MS | 4 | Y | Y | Y | Y | - |
|  |  |  |  |  | 2 | Y | Y | N | MS | 1 | Y | Y | Y | Y | - |
|  |  |  |  |  |  |  |  |  | MS | 2 | Y | Y | Y | Y | - |
|  |  |  |  |  |  |  |  |  | MS | 3 | Y | Y | Y | Y | - |
| 2 | 1 | Y | Y | N | 1 | N | N | N | None | - | - | - | - | - | a |
|  |  |  |  |  | 2 | Y | Y | N | MS | 1 | Y | Y | Y | Y | - |
| 3 | 1 | N | N | N | 1 | N | N | N | N/A | - | - | - | - | - | b |
| 4 | 1 | Y | Y | N | 1 | Y | Y | N | MS | 1 | Y | Y | Y | Y | - |
|  |  |  |  |  | 2 | Y | Y | N | MS | 1 | Y | N | Y | Y | - |
| 5 | 1 | N | N | N | 1 | N | N | N | None | - | - | - | - | - | a |
|  |  |  |  |  | 2 | N | N | N | None | - | - | - | - | - | a |
| 6 | 1 | N | N | N | 1 | N | N | N | None | - | - | - | - | - | a |
|  |  |  |  |  | 2 | N | N | N | None | - | - | - | - | - | a |
| 7 | 2 | Y | Y | Y | 1 | N | N | N | None | - | - | - | - | - | a |
|  |  |  |  |  | 2 | Y | Y | Y | B | 1 | N | - | - | - | c |
|  |  |  |  |  |  |  |  |  | B | 2 | Y | Y | - | - | - |
|  |  |  |  |  |  |  |  |  | MS | 3 | Y | Y | Y | Y | - |
| 8 | 2 | N | N | N | 1 | N | N | N | N/A | - | - | - | - | - | b |
| 9 | 2 | Y | Y | Y | 1 | Y | Y | N | MS | 1 | Y | Y | Y | Y | - |
|  |  |  |  |  |  |  |  |  | MS | 2 | Y | Y | Y | Y | - |
|  |  |  |  |  | 2 | Y | Y | Y | B | 1 | Y | Y | - | - | - |
|  |  |  |  |  |  |  |  |  | MS | 2 | Y | N | N | Y | - |
| 10 | 2 | Y | Y | Y | 1 | Y | Y | N | MS | 1 | Y | Y | Y | Y | - |
|  |  |  |  |  | 2 | Y | N | Y | B | 1 | Y | N | - | - | - |
| 11 | 2 | Y | Y | N | 1 | N | N | N | MS | 1 | N | - | - | - | c |
|  |  |  |  |  | 2 | Y | Y | N | MS | 1 | Y | Y | Y | Y | - |
| 12 | 2 | Y | Y | N | 1 | Y | Y | N | MS | 1 | Y | Y | Y | Y | - |
|  |  |  |  |  | 2 | Y | Y | N | MS | 1 | Y | Y | Y | Y | - |
| 13 | 2 | N | N | N | 1 | N | N | N | None | - | - | - | - | - | a |
|  |  |  |  |  | 2 | N | N | N | None | - | - | - | - | - | a |
| 14 | 2 | Y | N | Y | 1 | N | N | N | None | - | - | - | - | - | a |
|  |  |  |  |  | 2 | Y | N | Y | B | 1 | Y | N | - | - | - |
| 15 | 2 | Y | Y | N | 1 | Y | Y | N | MS | 1 | Y | N | N | N | - |
|  |  |  |  |  | 2 | Y | Y | N | MS | 1 | Y | N | N | N | - |
|  |  |  |  |  |  |  |  |  | MS | 2 | N | - | - | - | c |
| 16 | 2 | Y | Y | N | 1 | N | N | N | None | - | - | - | - | - | a |
|  |  |  |  |  | 2 | Y | Y | N | MS | 1 | Y | Y | N | Y | - |
| 17 | 3 | N | N | N | 1 | N | N | N | N/A | - | - | - | - | - | b |
| 18 | 3 | Y | Y | Y | 1 | N | N | N | None | - | - | - | - | - | a |
|  |  |  |  |  | 2 | Y | Y | Y | B | 1 | Y | Y | - | - | - |
|  |  |  |  |  |  |  |  |  | MS | 2 | Y | N | N | Y | - |
|  |  |  |  |  |  |  |  |  | MS | 3 | Y | Y | Y | Y | - |
|  |  |  |  |  |  |  |  |  |  |  |  |  |  |  |  |
| 19 | 3 | Y | Y | N | 1 | N | N | N | None | - | - | - | - | - | a |
|  |  |  |  |  | 2 | Y | Y | N | MS | 1 | Y | Y | Y | Y | - |
|  |  |  |  |  |  |  |  |  | MS | 2 | Y | Y | Y | N † | - |
|  |  |  |  |  |  |  |  |  | MS | 3 | Y | Y | Y | Y | - |
| 20 | 3 | Y | Y | Y | 1 | N | N | N | None | - | - | - | - | - | a |
|  |  |  |  |  | 2 | Y | Y | Y | B | 1 | Y | Y | - | - | - |
|  |  |  |  |  |  |  |  |  | MS | 2 | Y | Y | N | Y | - |
| 21 | 3 | Y | Y | N | 1 | Y | Y | N | MS | 1 | Y | Y | Y | Y | - |
|  |  |  |  |  | 2 | Y | Y | N | MS | 1 | Y | Y | Y | Y | - |
| 22 | 3 | N | N | N | 1 | N | N | N | None | - | - | - | - | - | a |
|  |  |  |  |  | 2 | N | N | N | None | - | - | - | - | - | a |
| 23 | 3 | Y | N | Y | 1 | Y | N | Y | B | 1 | Y | Y | - | - | - |
|  |  |  |  |  | 2 | N | N | N | None | - | - | - | - | - | a |
| **Total Included #** | | **16** | **14** | **7** | **1** | **8** | **7** | **1** | **MS** | **12** | **11** | **10** | **10** | **8** | **-** |
|  |  |  |  |  |  |  |  |  | **B** | **1** | **1** | **1** | **-** | **-** | **-** |
|  |  |  |  |  |  |  |  |  | **Total** | **13** | **12** | **11** | **10** | **8** | **-** |
|  |  |  |  |  | **2** | **15** | **13** | **6** | **MS** | **19** | **18** | **14** | **13** | **16** | **-** |
|  |  |  |  |  |  |  |  |  | **B** | **7** | **6** | **4** | **-** | **-** | **-** |
|  |  |  |  |  |  |  |  |  | **Total** | **26** | **24** | **18** | **13** | **16** | **-** |
|  |  |  |  |  | **Total** | **23** | **20** | **7** | **MS** | **31** | **29** | **24** | **23** | **24** | **-** |
|  |  |  |  |  |  |  |  |  | **B** | **8** | **7** | **5** | **-** | **-** | **-** |
|  |  |  |  |  |  |  |  |  | **Total** | **39** | **36** | **29** | **23** | **24** | **-** |

* TOMAC Sessions Activated “In Sleep Before/After Analyses” refers the exploratory evaluation of leg movements, arousals, and respiratory events in the period of sleep surrounding the awakening when the mid-sleep TOMAC session was activated. All mid-sleep sessions excluded from that analysis were due to insufficient duration of sleep prior to TOMAC activation per the “TOMAC Session Analysis” sub-section of the Methods section.

† n=2 mid-sleep TOMAC sessions were excluded from all analyses requiring a “before” TOMAC activation period (not only the analyses requiring a period of continuous sleep before TOMAC activation).

‡ Key for exclude reasons: a= No TOMAC sessions activated, b= Moderate-severe obstructive sleep apnea, c= Short TOMAC session duration during PSG recording (< 20 minutes).

Abbreviations: B= bedtime (TOMAC session activated prior to initial sleep onset), MS= mid-sleep (TOMAC session activated during awakening after initial sleep onset), N= no, PSG= polysomnography, Sess.= session, TOMAC= tonic motor activation, Y= yes.

Table S2: Demographics at enrollment for participants who only ran bedtime TOMAC sessions.

|  | Participants with only bedtime TOMAC |
| --- | --- |
| Number of participants | 2 |
| Age (y), mean (SD) | 45.9 (10.9) |
| % Female (n) | 0% (0) |
| Years since RLS Symptom Onset, mean (SD) | 3.8 (3.6) |
| Years since RLS Diagnosis, mean (SD) | 2.9 (2.9) |
| Years since RLS Starting Prescription RLS Medication, mean (SD) | 2.9 (2.9) |
| Obstructive Sleep Apnea Diagnosis, % (n) | 0% (0) |
| Refractory RLS Medications, % (n) |  |
| Dopamine Agonist | 50.0% (1) |
| Alpha-2-Delta Ligand | 100% (2) |
| Both | 50.0% (1) |
| Current RLS Medications, % (n) |  |
| None | 0% (0) |
| Dopamine Agonist | 50.0% (1) |
| Alpha-2-Delta Ligand | 50.0% (1) |
| Benzodiazepines | 0% (0) |
| Opioids | 0% (0) |
| Total IRLS Score, mean (SD) | 21.0 (4.2) |

Demographics at enrollment for participants who only ran bedtime (not mid-sleep) TOMAC sessions and are only included in the supplementary analysis of sleep onset latency after activation of a bedtime TOMAC session. Abbreviations: RLS= Restless Legs Syndrome, IRLS= International Restless Legs Syndrome Study Group Rating Scale, SD= standard deviation.

Table S3: Full-night polysomnographic parameters for each night where a mid-sleep TOMAC session was activated and included in the analyses (n= 14 participants, 20 PSG nights).

| PSG Parameter | Mean (SD) |
| --- | --- |
| Sleep Architecture |  |
| Total Recording Time (min, TRT) | 423.2 (65.4) |
| Total Sleep Time (min, TST) | 311.3 (79.9) |
| Sleep Latency (min) | 14.5 (12.6) |
| Stage REM Latency (min) | 145.0 (91.9) |
| Awakenings (number/h) | 4.2 (1.8) |
| Sleep Efficiency (TST/TRT, %) | 73.4 (14.2) |
| Wake After Sleep Onset (min) | 88.3 (55.9) |
| Sleep Stage N1 (% of TST) | 3.2 (2.0) |
| Sleep Stage N2 (% of TST) | 56.0 (12.8) |
| Sleep Stage N3 (% of TST) | 21.5 (10.1) |
| Sleep Stage REM (% of TST) | 19.4 (7.6) |
| Leg Movements During Sleep (LMS) |  |
| LMS Index (number LMS/h) | 40.8 (40.3) |
| PLMS Index (number PLMS/h) | 34.1 (40.4) |
| PLMA Index (number PLMS associated with arousal/h) | 6.9 (9.1) |
| PLMS Sequences (number) | 10.0 (12.6) |
| Periodicity Index | 0.44 (0.32) |
| Arousal and Respiratory Event Indices |  |
| Arousal Index (number/h) | 12.8 (11.2) |
| Respiratory Effort-related Arousals (RERA) Index (number/h) | 3.6 (3.5) |
| Apnea Hypopnea Index (AHI, number/h) | 3.5 (4.2) |
| Respiratory Disturbance Index (number/h) | 7.1 (5.7) |

Abbreviations: PLMA= periodic leg movements during sleep associated with arousal, PLMS= periodic leg movements during sleep, REM= rapid eye movement, TOMAC= tonic motor activation.

## Appendix S2: Compatibility of TOMAC with initial sleep onset at bedtime

TOMAC sessions activated at bedtime were analyzed to further evaluate the compatibility of TOMAC with initial sleep onset. Sleep initiation was demonstrated during 71.4% (5 of 7) of bedtime TOMAC sessions. Mean±SD sleep onset latency from TOMAC activation was 21.5±17.4 minutes (median 15.6) and latency from lights out to sleep onset was 29.9±21.3 minutes (median 29.5). Participants utilized similar TOMAC stimulation intensity at bedtime to the mid-sleep TOMAC sessions (mean±SD stimulation intensity= 26.3±3.9 mA).

## Supplementary Figures

**
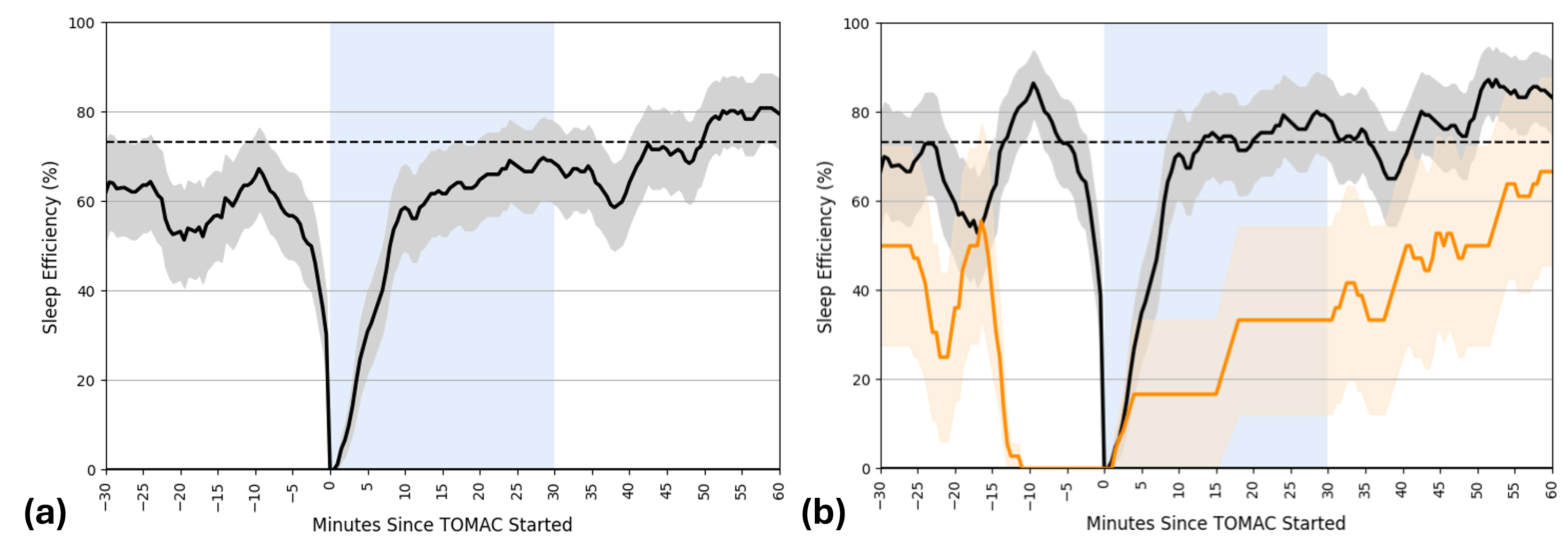
**

Figure S1: Sleep efficiency preceding and following mid-sleep awakening and TOMAC activation.

For (a) all mid-sleep TOMAC sessions with sufficient data prior to TOMAC activation (n=27 sessions) and (b) comparison between mid-sleep sessions started ≤ 10-minutes after awakening (black, n=21 sessions) and sessions started > 10-minutes after awakening (orange, n=6 sessions). The programmed 30-minute TOMAC duration is shaded blue. Sleep efficiency weighted average for the full night for sessions included in this analysis (73.3%) is shown by the dashed line for reference. Shaded error bands correspond to ± SEM. Abbreviations: TOMAC= tonic motor activation.

**
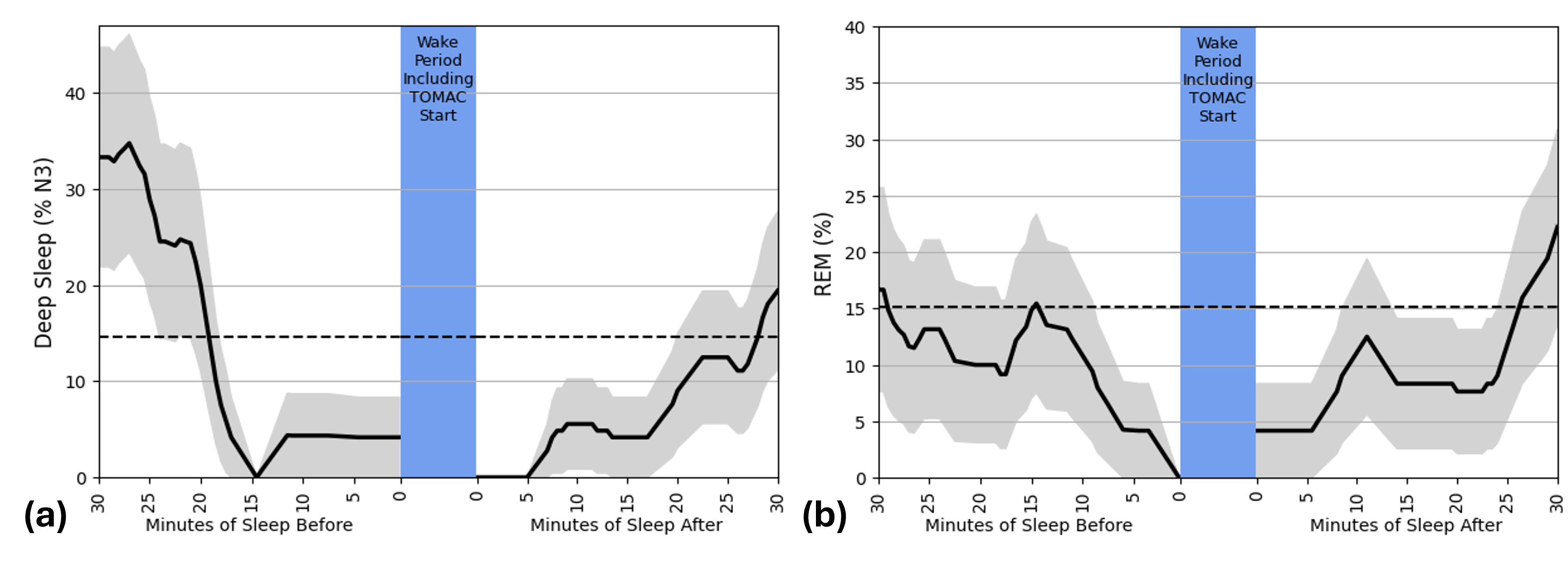
**

Figure S2: Transitions in sleep architecture preceding and following mid-sleep TOMAC activation.

The proportion of mid-sleep sessions (normalized to the awakening to activate TOMAC shown in blue, n=24 sessions) where the 30-second epoch was scored as (a) N3 (full night average: 14.6%) and (b) REM (full night average: 15.1%). The weighted average for the full night is shown by the dashed line for reference and shaded error bands correspond to ± SEM. Abbreviations: REM= rapid eye movement, TOMAC= tonic motor activation.


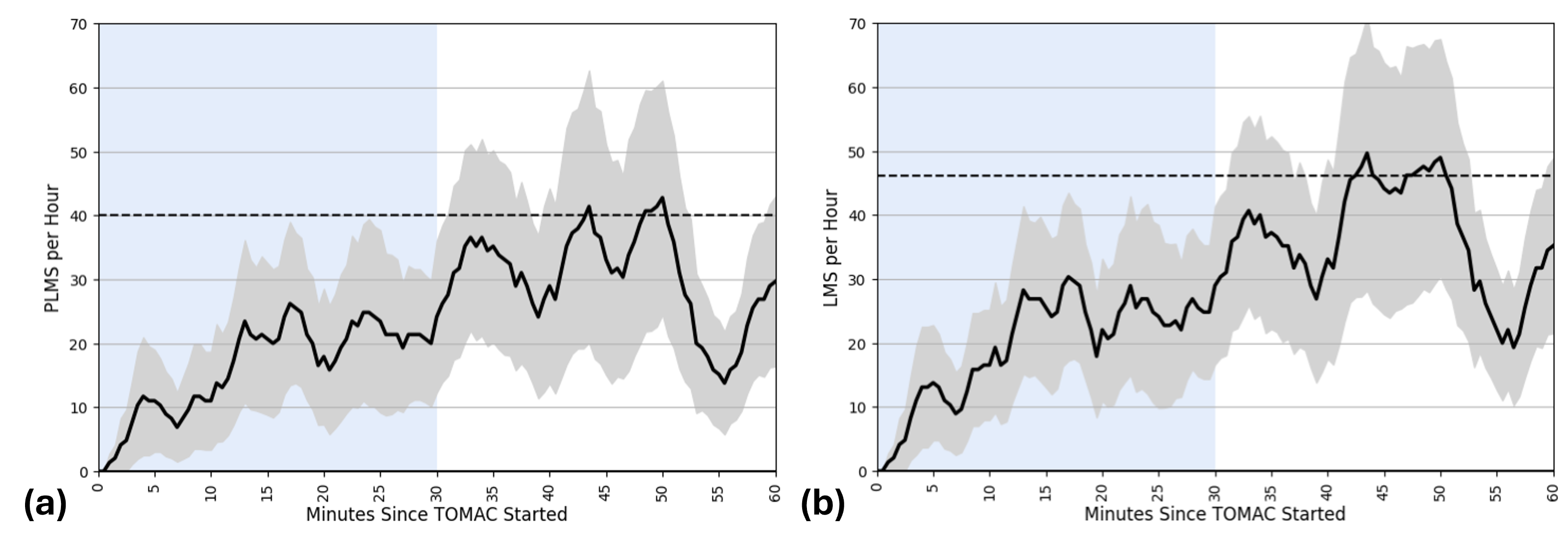


Figure S3: Leg movements remain mostly lower than average in the hour following mid-sleep TOMAC activation.

(a) PLMS and (b) LMS in the 60-minutes since the mid-sleep TOMAC session was activated (n=29 sessions). The programmed 30-minute TOMAC duration is shaded blue. The weighted average for the full night is shown by the dashed line for reference (40.1 PLMS/h, 46.2 LMS/h). Shaded error bands correspond to ± SEM. Abbreviations: LMS= leg movements during sleep, PLMS= periodic leg movements during sleep, TOMAC= tonic motor activation.


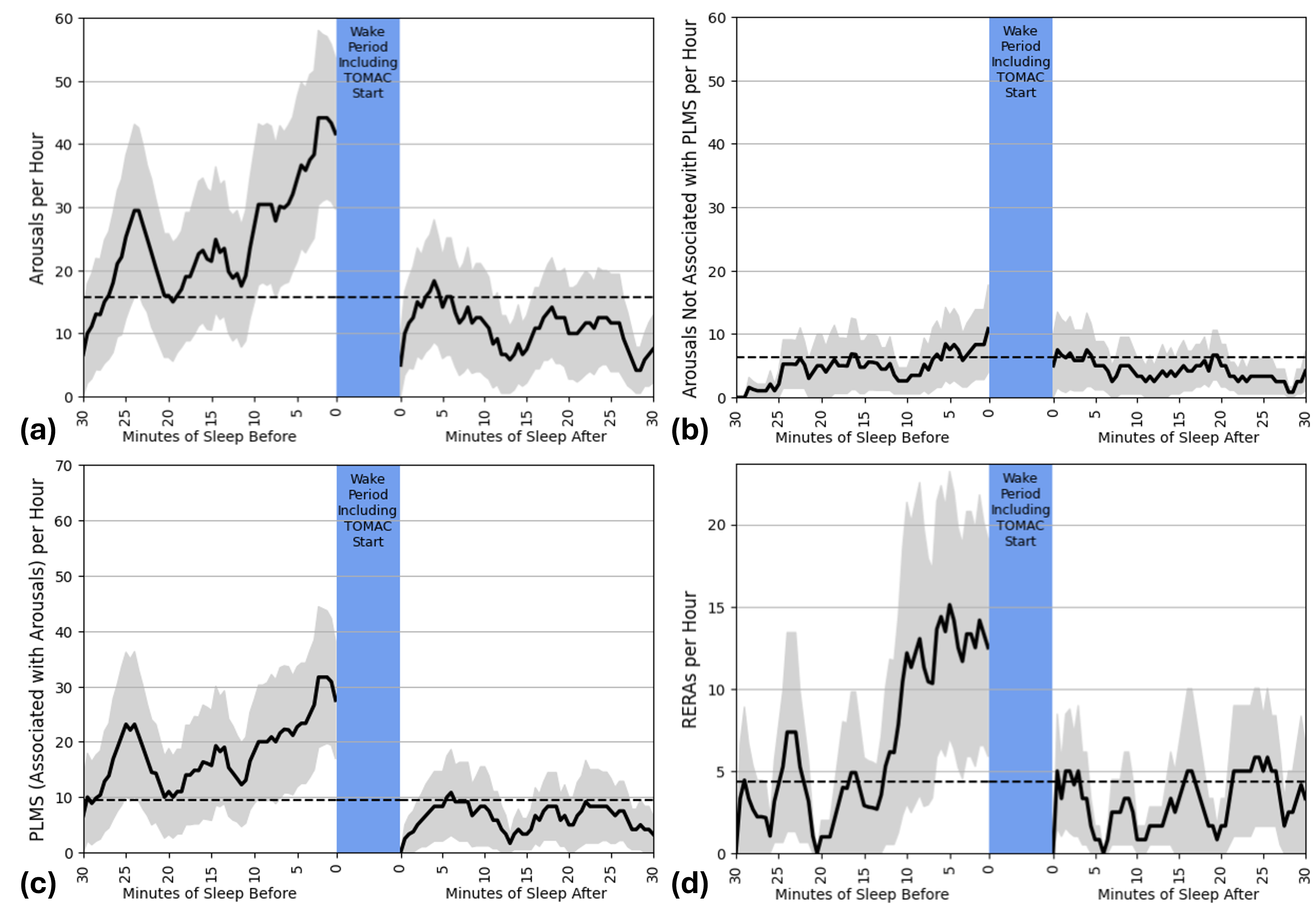


Figure S4: Changes related to arousals in the 30-minutes of preceding and following mid-sleep TOMAC activation.

Changes in (a) total arousals per hour, (b) arousals not associated with PLMS per hour, (c) PLMS associated with arousals (PLMA) per hour, and (d) RERAs (n=24 sessions). The period of awakening when TOMAC was activated is shaded blue. The weighted average for the full night for each metric is shown by the dashed line for reference. Shaded error bands correspond to ± SEM. Abbreviations: PLMS= periodic leg movements during sleep, RERAs= respiratory effort-related arousals, TOMAC= tonic motor activation.

## Appendix S3: PSG First Night Effect

To evaluate the effect of adaptation to the PSG recording, we performed a sensitivity analysis to evaluate differences between mid-sleep TOMAC sessions from the first PSG with TOMAC (n=11 sessions) and the second PSG with TOMAC (n=18 sessions). TOMAC stimulation intensity was lower for sessions during the first PSG (mean±SD= 22.5±3.7 mA) than the second (27.4±5.3 mA, p=0.014). TOMAC session characteristics, sleep efficiency, N3 and REM sleep percentages before and after TOMAC were similar across each of the PSG nights, suggesting that differences in stimulation intensity between nights was unlikely to have impacted the sleep re-initiation and depth outcomes. Movement, arousal, and respiratory measures prior to mid-sleep TOMAC activation were similar between nights. PLMS/h (p=0.022), and arousal index (p=0.038) were lower following mid-sleep TOMAC during the second PSG than the first PSG, while PLMA/h (p=0.256) and RERAs/h (p=0.242) were similar following TOMAC between nights. When comparing sleep before and after starting TOMAC, the rate of PLMA and total arousals decreased for the second PSG but not the first PSG night, and rates of PLMS, LMS, and RERAs had similar changes across PSG nights.
